# Management and investigation of a *Serratia marcescens* outbreak in a neonatal unit in Switzerland – the role of hand hygiene and whole genome sequencing – R1, ARIC-D-17-00143

**DOI:** 10.1186/s13756-017-0285-x

**Published:** 2017-12-11

**Authors:** Walter Zingg, Isabelle Soulake, Damien Baud, Benedikt Huttner, Riccardo Pfister, Gesuele Renzi, Didier Pittet, Jacques Schrenzel, Patrice Francois

**Affiliations:** 1 Infection Control Program and WHO Collaborating Center for Patient Safety, |University of Geneva Hospitals, 4 Rue Gabrielle Perret-Gentil, 1211, 14 Geneva, Switzerland; 2 Genomic Research Laboratory, Division of Infectious Diseases, University of Geneva Hospitals, Geneva, Switzerland; 3 Division of Infectious Diseases, University of Geneva Hospitals and Faculty of Medicine, Geneva, Switzerland; 4 Neonatal Intensive Care Unit, Department of Paediatrics, University of Geneva Hospitals, Geneva, Switzerland; 5 Bacteriology Laboratory, Department of Genetics and Laboratory Medicine, University of Geneva Hospitals, Geneva, Switzerland

**Keywords:** Neonates, Neonatal intensive care unit, *Serratia Marcescens*, Outbreak, Hand hygiene, Isolation, Infection control, Whole genome sequencing, Cross-transmission, Healthcare-associated infection

## Abstract

**Background:**

Many outbreaks due to *Serratia marcescens* among neonates have been described in the literature but little is known about the role of whole genome sequencing in outbreak analysis and management.

**Methods:**

Between February and March 2013, 2 neonates and 2 infants previously hospitalised in the neonatal unit of a tertiary care centre in Switzerland, were found to be colonised with *S. marcescens*. An investigation was launched with extensive environmental sampling and neonatal screening in four consecutive point prevalence surveys between April and May 2013. All identified isolates were first investigated by fingerprinting and later by whole genome sequencing. Audits of best practices were performed and a hand hygiene promotion programme was implemented.

**Results:**

Twenty neonates were colonised with *S. marcescens*. No invasive infection due to *S. marcescens* occurred. All 231 environmental samples were negative. Hand hygiene compliance improved from 51% in April 2013 to 79% in May 2013 and remained high thereafter. No *S. marcescens* was identified in point prevalence surveys in June and October 2013. All strains were identical in the fingerprinting analysis and closely related according to whole genome sequencing.

**Conclusions:**

Improving best practices and particularly hand hygiene proved effective in terminating the outbreak. Whole genome sequencing is a helpful tool for genotyping because it allows both sufficient discrimination of strains and comparison to other outbreaks through the use of an emerging international database.

## Background

*Serratia marcescens* has long been recognized as an important pathogen in neonatal intensive care units (NICUs). It is the third most common pathogen identified in published NICU outbreaks [1], and it has been found to account for 15% of all culture-positive nosocomial infections in this setting [2]. The large number of outbreak reports underestimates the true occurrence of *S. marcescens* in neonatology units and NICUs. According to the results of the mandatory surveillance of healthcare-associated infections (HAIs) in very low birth weight infants in Germany from 2006 to 2011, at least one to two *Serratia* outbreaks per year are expected for Germany alone [3].

*S. marcescens* causes a wide range of clinical manifestations in neonates, from asymptomatic colonization to infections such as urinary tract infections, pneumonia, sepsis or meningitis [4, 5]. Risk factors for *Serratia* spp. acquisition by neonates are related to immaturity, prolonged hospital stay, antibiotic use, and mechanical ventilation [4, 6].

The objective of this outbreak report was to summarize the investigation and successful management of a *S. marcescens* outbreak in neonates and to investigate the contribution of using whole genome sequencing. This report follows the ORION (Outbreak Reports and Intervention Studies Of Nosocomial infection) statement [7].

## Methods

### Setting

The University of Geneva Hospitals (HUG), are a 1′800-bed primary and tertiary care center with about 47,000 admissions accounting for 660,000 patient-days per year. At the time of the outbreak it offered 17 places in the NICU, and 12 places in the geographically separate pediatric intensive care unit (PICU), where neonates are cared for when mechanical ventilation is needed. Neonates who are clinically stable but need prolonged stay for non-medical reasons are transferred to the unit for child development (UCD).

### Outbreak

**Fig. 1:**
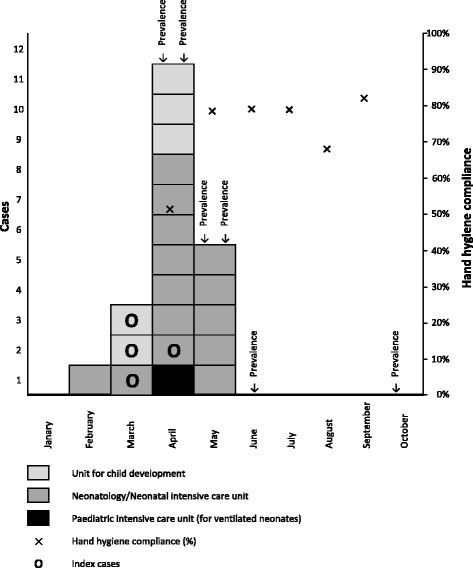
Epidemic curve – *Serratia marcescens* outbreak at the University of Geneva Hospitals, 2013

**Fig. 2:**
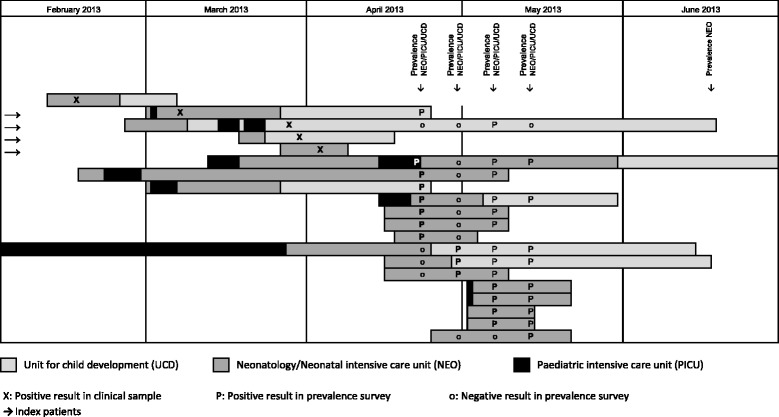
Cases – *Serratia marcescens* outbreak at the University of Geneva Hospitals, 2013

The outbreak started in February 2013 and ended in June 2013 (Fig. 1). Between February and March 2013, two neonates in the NICU and 2 infants in the UCD were found with *S. marcescens* (2 vascular catheters, 2 eye swabs, and 1 urine sample). Given the organizational ties and the patient flow between NICU, PICU, and UCD, an investigation was launched by a first point prevalence survey in the three units (Fig. 2) on 23 April 2013. Eight out of 41 screened children were identified as cases in this survey, one in the PICU, 3 in the UCD and 4 in the NICU. All new cases were neonates with a present or past history of stay in the NICU. Based on these findings, we focused further activity on the NICU with extensive environmental sampling and neonatal screening during 4 consecutive prevalence surveys.

### Outbreak management

Audits of best practices in the use of surface disinfectants, ointments and cosmetic products were performed, as well as weekly direct hand hygiene observations (439 opportunities in total). An intensive hand hygiene promotion programme was offered to the staff working in the NICU: daily presence in the NICU, education and training, and weekly hand hygiene audits with individual feedback.

### Microbiological investigation/genotyping

In 2013, the genomes of the *S. marcescens* isolates obtained in the prevalence surveys were tested by a commercial fingerprinting assay (DiversiLab®, Biomerieux, France). In 2016, the genomes *S. marcescens* isolates were fully sequenced using an Illumina HiSeq 2500 sequencer as previously described [8].

## Results

A total of 232 neonatal screenings (117 stool samples, 115 nasal swabs) [9] were performed. In addition to the 4 index cases and the 8 cases identified in the first prevalence survey, an additional three, four and one cases were identified in the second, third and fourth prevalence surveys in April and May, respectively (Fig. 2). The proportions of positive rectal and nasal swabs were 11.1% (13/117) and 6.1% (7/115), respectively. Five infants had a positive result for both rectal and nasal swabs. All cases were preterm-, and seven were very-low-birth weight infants. No new *S. marcescens* cases were identified until October 2016, including two further prevalence surveys, which were performed in June and October. There was no invasive infection due to *S. marcescens*, neither during the outbreak nor until end of December 2013. A total of 231 environmental samplings were performed (Table 1). All tested products, materials and surfaces were negative for *S. marcescens*. Hand hygiene compliance improved from 51% in April 2013 to 79% in May 2013 following the promotion programme. Compliance remained above 65% in the following months (Fig. 1).

**Table 1:** Products, materials and surfaces tested for microbiological growth – *Serratia marcescens* outbreak at the University of Geneva Hospitals, 2013

Products, materials and surfaces	N (%)
Hand and skin disinfectants	66 (28.6)
Hypochlorite solution 225 ppm to disinfect pacifiers and nipple shields	25 (10.8)
Ointments and body lotions	25 (10.8)
Computer keyboards	20 (8.7)
Medicated and non-medicated soaps	20 (8.7)
Bowls for body care	17 (7.4)
Water from incubators	11 (4.8)
Tap water	10 (4.3)
Tubs	9 (3.9)
Sinks	9 (3.9)
Water from CPAP^a^ tubes	6 (2.6)
Other materials or surfaces	13 (5.6)
Total	231 (100)

There was no growth for *S. marcescens* on any of the tested products, materials or surfaces

^a^ *CPAP* continuous positive airway pressure

**Fig. 3:**
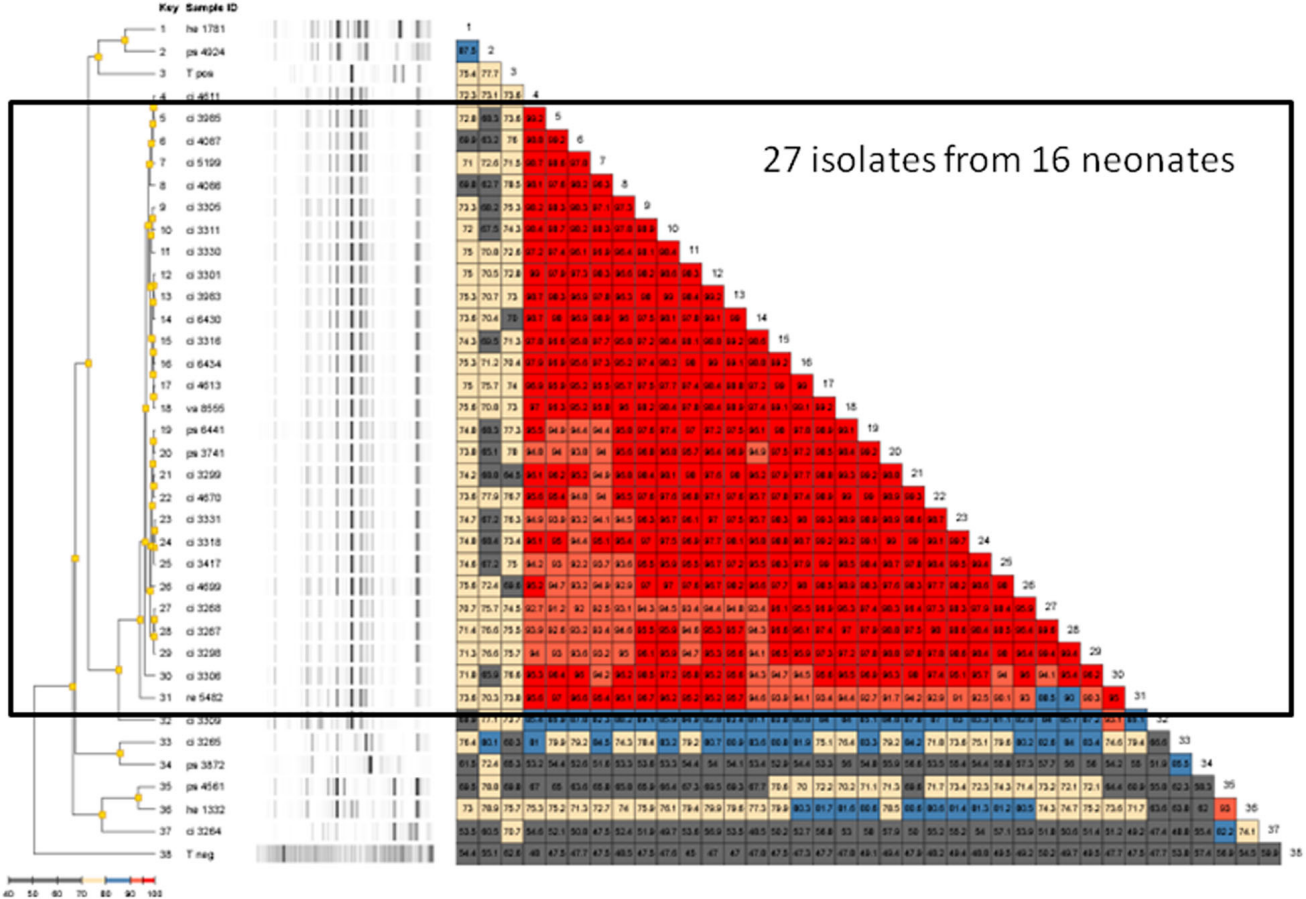
Fingerprinting of *S. marcescens* strains using the DiversiLab® kit – *Serratia marcescens* outbreak at the University of Geneva Hospitals, 2013

**Fig. 4:**
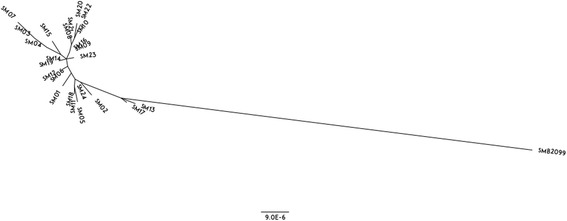
Whole genome SNP based phylogenetic analysis of *Serratia marcescens* strains – *Serratia marcescens* outbreak at the University of Geneva Hospitals, 2013

Fingerprinting of 24 isolates from 16 neonates showed identical strains (Fig. 3). Whole genome sequencing was carried out on the set of 24 *S. marcescens* strains (Sm01-Sm24) belonging to the outbreak. The sequencing on an Illumina HiSeq produced on average a total of 13 million 150 bp reads per sample, exhibiting very high theoretical coverage values (between 300 and 900 fold). Assembly was achieved using SPAdes 3.9.0 software, after read quality trimming and filtering was applied to the raw reads. The assembly produced very similar results for each strain from the outbreak, resulting in an average genome size of 5′080’124 bp with a standard deviation of 1097 bp. This shows extreme similarity between the strains of the outbreak. Moreover, pairwise comparisons using MUMmer software were performed between each couple of strains and showed similarity above 99.999% [10]. Figure 4 shows an unrooted tree displaying all the isolates from the outbreak (Sm01-24), and one representative strain isolated in Germany during a SM outbreak in 2016 (SMB2099) [11].

## Discussion

This report suggests that focusing on and improving hand hygiene is effective in terminating a *S. marcescens* outbreak among neonates. Whole genome sequencing is a useful tool for outbreak investigation because it is more discriminative than standard fingerprinting tests and allows benchmarking with other outbreaks thanks to a growing gene bank.

The source of *S. marcescens* outbreaks in NICUs is rarely identified. A review summarizing 48 NICU outbreaks reported an identified source in only 40% (19/48), the most frequent being a colonized or infected index patient (8/48), followed by equipment for patient care (6/48), an (unspecified) environment (4/48), and food (1/48) [12]. Colonized or infected patients are thought to represent the most important reservoir of *S. marcescens* outbreaks in NICUs [4, 13]. In the currently described situation, the investigation revealed two additional NICU cases prior to the outbreak. A mother of one of them hadan amnion infection syndrome due to *S. marcescens* three months before the start of the outbreak, and thus, may have been the most likely source [12]. In the literature, healthcare workers were never identified as a source, but few studies investigated this specifically [14]. Like in our study, extensive environmental screening rarely yielded positive results in the reported outbreaks, suggesting that environmental contamination may play a limited role.

The clinical implication of colonization with *S. marcescens* in neonates and its implication in outbreaks is not always clear. A number of studies reported that *S. marcescens* is rather commonly isolated in stool of preterm neonates within the first weeks of life [6, 15, 16]. However, the pathogen was not isolated consistently in preterm neonates either [17], and *S. marcescens* has not been isolated in healthy term babies [18]. Thus, while the finding of *S. marcescens* in neonatal stool can be considered a regular event in NICUs, the pathogen per se is not part of the normal early gut flora of healthy newborns in the community. Once the intestines of a neonate are colonized with *S. marcescens* from the (pathologic) outside, they can become the “source” of an outbreak. This makes control of *Serratia* outbreaks very difficult, and underlines the importance of hands in the transmission of the pathogen and the possible successful management of an outbreak.

Already in 1981, contaminated hand-washing brushes were reported to be implicated in a *S. marcescens* outbreak, indirectly pointing to the pivotal role of hand hygiene in the spread of the pathogen [19]. Contaminated soaps were identified in another report [20]. Interventions to control outbreaks included always both hand hygiene and environmental control [9, 21–28]. Given that all environmental samples were negative in our outbreak investigation and work had been done in the past to dedicate equipment, drugs, ointments and other cosmetic products to individual neonates (avoiding multivials) [29], our intervention focused on hand hygiene improvement. This was all the more justified because a decrease of hand hygiene compliance had been identified as part of the investigation; hand hygiene compliance was 72% in the year before the outbreak.

Whole genome sequencing technology emerged as a powerful tool to identify clonality among outbreak isolates. The genome of a *S. marcescens* strain that sparked an outbreak among infants in Germany was reported very recently [11, 12]. Comparison with our index case revealed a moderate difference around 200 SNPs. Other isolates identified from our institution and not related to the outbreak were sequenced and the number of SNPs was higher than 100′000 (data not shown).

There are limitations in our outbreak investigation. First, environmental sampling was large but we did not consistently use neutralizing agents for testing the different cosmetic products, resulting in potential underreporting. However, cosmetic products were strictly confined to the neonates and no multivials were in use. A positive finding of a product with a case would not have been prove of the product being the source. Second, we did not produce formal records from the audits other than hand hygiene.

## Conclusions

Improving best practice and particularly hand hygiene are effective in terminating the outbreak. This highlights the role of hand hygiene in the cause but also in the successful management of *S. marcescens* outbreaks in neonates. Whole genome sequencing is a helpful tool for genotyping because it allows both sufficient discrimination of strains within the outbreak and comparison to other outbreaks through an emerging international database.

## Abbreviations

HAI: Healthcare-associated infection
HUG: University of Geneva hospitals
NICU: Neonatal intensive care unit
ORION: Outbreak reports and intervention studies of nosocomial infection
PICU: Pediatric intensive care unit
SNP: Single nucleotide polymorphism
UCD: Unit for child development
