# Correction to: Management and investigation of a *Serratia marcescens* outbreak in a neonatal unit in Switzerland – the role of hand hygiene and whole genome sequencing

**DOI:** 10.1186/s13756-017-0295-8

**Published:** 2018-01-16

**Authors:** Walter Zingg, Isabelle Soulake, Damien Baud, Benedikt Huttner, Riccardo Pfister, Gesuele Renzi, Didier Pittet, Jacques Schrenzel, Patrice Francois

**Affiliations:** 1 Infection Control Program and WHO Collaborating Center for Patient Safety, University of Geneva Hospitals, 4 Rue Gabrielle Perret-Gentil, 1211, 14 Geneva, Switzerland; 2 Genomic Research Laboratory, Division of Infectious Diseases, University of Geneva Hospitals, Geneva, Switzerland; 3 Division of Infectious Diseases, University of Geneva Hospitals and Faculty of Medicine, Geneva, Switzerland; 4 Neonatal Intensive Care Unit, Department of Paediatrics, University of Geneva Hospitals, Geneva, Switzerland; 5 Bacteriology Laboratory, Department of Genetics and Laboratory Medicine, University of Geneva Hospitals, Geneva, Switzerland

## Correction

The original article [1] contained an error whereby an erroneous article ID was displayed at the end of the title; the original article has now been corrected to remove this ID.

In addition, this error was mistakenly carried forward by the production team that handled this article, and thus, was not the fault of the authors.
